# An uncertain model-based approach for identifying dynamic protein complexes in uncertain protein-protein interaction networks

**DOI:** 10.1186/s12864-017-4131-6

**Published:** 2017-10-16

**Authors:** Yijia Zhang, Hongfei Lin, Zhihao Yang, Jian Wang, Yiwei Liu

**Affiliations:** College of Computer Science and Technology, Dalian University of Technology, Dalian, 116023 China

## Abstract

**Background:**

Recently, researchers have tried to integrate various dynamic information with static protein-protein interaction (PPI) networks to construct dynamic PPI networks. The shift from static PPI networks to dynamic PPI networks is essential to reveal the cellular function and organization. However, it is still impossible to construct an absolutely reliable dynamic PPI networks due to the noise and incompletion of high-throughput experimental data.

**Results:**

To deal with uncertain data, some uncertain graph models and theories have been proposed to analyze social networks, electrical networks and biological networks. In this paper, we construct the dynamic uncertain PPI networks to integrate the dynamic information of gene expression and the topology information of high-throughput PPI data. The dynamic uncertain PPI networks can not only provide the dynamic properties of PPI, which are neglected by static PPI networks, but also distinguish the reliability of each protein and PPI by the existence probability. Then, we use the uncertain model to identify dynamic protein complexes in the dynamic uncertain PPI networks.

**Conclusion:**

We use gene expression data and different high-throughput PPI data to construct three dynamic uncertain PPI networks. Our approach can achieve the state-of-the-art performance in all three dynamic uncertain PPI networks. The experimental results show that our approach can effectively deal with the uncertain data in dynamic uncertain PPI networks, and improve the performance for protein complex identification.

**Electronic supplementary material:**

The online version of this article (10.1186/s12864-017-4131-6) contains supplementary material, which is available to authorized users.

## Background

Over the past decade, yeast two-hybrid, mass spectrometry and other high-throughput experimental have generated a mass of protein-protein interaction (PPI) data. Such PPI data construct the large-scale PPI networks for many organisms. Great efforts have been made to understand organizational principles underlying PPI networks. Many cellular principles have been uncovered by analysis of these networks, such as the scale-free topology [1], disassortativeness [2] and modularity [3].

A protein complex consists of a group of proteins and multiple PPIs at the same time and place, forming single multi-molecular machinery [4]. Since most proteins are only functional after assembly into protein complexes, protein complexes are critical in many biological processes [5]. Over the past decade, great effort has been made to detect complexes on the PPI networks. The Molecular Complex Detection (MCODE) algorithm proposed by Bader and Hogue is the first time to exploit computational methods to identify complexes based on PPI networks [6]. Markov Clustering (MCL) [7] can use random walks to identify based on PPI networks. Liu et al. [8] propose Maximal Cliques Clustering (CMC) to predict complexes from large PPI networks. Based on the core-attachment structural feature [9], Leung et al. [10] propose CORE algorithm to identify protein-complex cores by calculating the *p*-values for all pairs of proteins. Similarly, Wu et al. [11] present COACH algorithm to identify protein complexes, which detects the core structure and attachments of complex respectively. Nepusz et al. [12] propose ClusterONE algorithm which effectively improves the performance to identify the overlapping complexes. Zhang et al. [13] propose CSO algorithm to predict complexes by integrating GO data and PPI networks.

A protein complex is formed by a group of proteins at the same time, which interacted with each other by associated polypeptide chains. However, modeling biology systems as static PPI networks will lose the temporal information. It is necessary to construct dynamic PPI networks for both identifing protein complexes and further understanding molecular systems. Since gene expression data is helpful to analyze the temporal information of proteins, some studies [14–18] have used gene expression data to construct dynamic PPI networks and reveal the dynamic character of PPI networks. For example, Faisal et al. [14] predict human aging-related genes by integrating aging-related gene expression data with human PPI data. Wang et al. [15] construct dynamic PPI networks and detect complex by exploiting gene expression data and PPI data.

Another issue in complexes identification is PPI networks contain much noise data including false positive and false negative rates [16]. Some studies have been proposed to improve the reliability of PPI networks [17]. Using uncertain graph model to deal with such PPI networks is more reasonable than traditional graph model. Uncertain model have been applied to analyze social networks, electrical networks and biological networks. Recently, Zhao et al. [18] use uncertain model to detect protein complexes in static PPI networks. Nonetheless, few studies apply uncertain model to analyze dynamic PPI networks.

In this study, we firstly construct dynamic uncertain PPI networks (DUPN) by integrating gene expression and PPI data. The active time point and the existence probability of each protein is calculated based on gene expression data. The existence probability of each PPI is calculated based on the topological property of high-through PPI data. We then attempt to use uncertain graph model to identify the protein complexes in DUPN, and propose a clustering algorithm named CDUN. Finally, we evaluate our method in different datasets and the experimental results show that our method achieves the state-of-the-art performance for complex identification.

## Methods

In this section, we introduce how to integrate the gene expression data with the PPI data to construct the DUPN, and then describe the clustering algorithm CDUN for identify protein complexes based on the DUPN in details.

### Active time points and probability of proteins

In a living cell, proteins and PPIs are not static but changing over time [19]. The gene expression is useful to analyze the temporal information of the proteins. In recent years, some studies [15, 20, 21] have use gene expresstion data to calculate the active time points of proteins.

The gene expression data consist of *n* time point profiles. Let *G*_*i*_ *(p)* denote the gene *p* expression value at *i* time point. Let *α*(*p*)and *σ*(*p*)be the arithmetic mean and the standard deviation (SD) of Gi(p), respectively.

1 $$ \alpha (p)=\frac{\sum_{i=1}^n{G}_i(p)}{n} $$

2 $$ \sigma (p)=\sqrt{\frac{\sum_{i=1}^n{\left({G}_i(p)-\alpha (p)\right)}^2}{n-1}} $$

Let *X* be a real random variable of normal distribution *N*(*α, σ* ^2^). For any *k > 0*, P{|*X-α*| < *kσ*} = 2Φ(*k*)-1, where Φ(*.*) is the distribution function of the standard normal law [15, 20].

In this study, we use the Eqs. (3) and (4) to calculate protein active probability at the different time points.

3 $$ Ge\_{thresh}_k(p)=\alpha (p)+k\cdotp \sigma (p)\cdotp \left(1-\frac{1}{1+{\sigma}^2(p)}\right) $$

4 $$ {\mathrm{Pr}}_i(p)=\left\{\begin{array}{ll}0.99\hfill & if\ {G}_i(p)\ge Ge\_{thresh}_3(p)\hfill \\ {}0.95\hfill & if\  Ge\_{thresh}_3(p)>{G}_i(p)\ge Ge\_{thresh}_2(p)\hfill \\ {}0.68\hfill & if\  Ge\_{thresh}_2(p)>{G}_i(p)\ge Ge\_{thresh}_1(p)\hfill \\ {}0\hfill & if\ {G}_i(p)< Ge\_{thresh}_1(p)\hfill \end{array}\right. $$

We use the Eq. (3) to calculate the *k*-sigma (*k* = 1,2,3) threshold for the gene *p*. *Ge_thres*_*k*_ is determined by the values of *α*(*p*),*σ* ^2^(*p*)and *k* (the times of sigma). If *σ* ^2^(*p*)is very low, it indicates that the fluctuation of the expression curve of gene *p* is also very small and the value of G_i_(*p*) tends to be very close to *α*(*p*). In this case, the value of *Ge_thresh*_*k*_ is close to *α*(*p*). If *σ* ^2^(*p*)is very high, it indicates much noise in the gene expression data of the gene *p*. In this case, the value of *Ge_thresh*_*k*_ is close to *α*(*p*) + *k* · *σ*(*p*). In the Eq. (3), the range of *k* (the times of sigma) is in (0, 3), and 3 is the maximum times of sigma. The larger *k* is, the higher *Ge_thresh*_*k*_ gets. A higher value of *Ge_thresh*_*k*_ indicates that using more strict rules to identify the active time point of a protein [20].

We use the Eq. (4) to calculate the active probability of a protein in the *i* time point. Thus, the protein active probability contains four levels (0.99, 0.95, 0.68 and 0) based on the sigma rules (P{|*X-α*| < *σ*} *≈* 0.6827, P{|*X-α*| < 2*σ*} *≈* 0.9545 and P{|*X-α*| < 3*σ*} *≈* 0.9973) [15, 20].

### Construction of DUPN

**Fig. 1:**
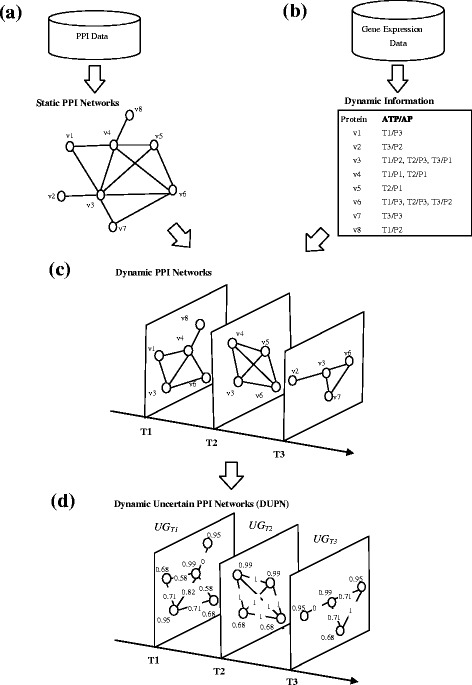
An illustration example of DUPN construction. **a** construction of static PPI networks. **b** calculation of dynamic information. ATP and AP denote active time points and active probability, respectively. **c** dynamic PPI networks. **d** dynamic uncertain PPI networks (DUPN)

Figure 1 shows an illustration example of the DUPN construction. Firstly, we use the PPI data to construct the static PPI networks in Fig. 1a. Secondly, we use gene expression data to calculate the active time points and the probability of each in Fig. 1b. In this study, the active probability only include three values *P1* = 0.99, *P2* = 0.95 and *P3* = 0.68 based on the Eq. (4). Although a PPI imply physical contact between two proteins, it does not mean that the interaction occur in a cell at any time [22]. The real PPI networks are changing during the lifetime of a cell, because the active time points of proteins are different. Thirdly, we can inject the static PPI networks into a series of PPI subnetworks based on the dynamic information of the proteins in Fig. 1c. These PPI subnetworks associated with the different active time points construct a dynamic PPI network. All proteins in the PPI subnetworks *Ti* are active with an active probability at *Ti* time point. Finally, we assign an uncertain value to each protein and PPI in the dynamic PPI networks to construct the DUPN in Fig. 1d. In this way, we can distinguish the uncertain level of both protein and PPI in the DUPN. The existence probability of each protein is the active probability calculated based on Eq. (4). Zhao et al. [18] proposed a method to calculate the existence probability of PPI based on the topology structure of the PPI networks. In this study, we use the same method to calculate the existence probability of each PPI on the Fig. 1d based on the topology structure of the PPI subnetworks in the Fig. 1c. The existence probability between the two proteins *v*_*j*_ and *v*_*k*_ is defined as follows:

5 $$ {\mathrm{Pr}}_{Ti}\left({v}_j,{\mathrm{V}}_{\mathrm{k}}\right)=\left\{\begin{array}{ll}\sqrt{\frac{{\left|{N}_j\cap {N}_k\right|}^2}{\left(\left|{N}_j\right|-1\right)\cdotp \left(\left|{N}_k\right|-1\right)},}\hfill & \left|{N}_j\right|>1\kern0.5em \mathrm{and}\kern0.5em \left|{N}_k\right|>1\hfill \\ {}0,\hfill & \left|{N}_j\right|=1\kern1em \mathrm{or}\kern0.5em \left|{N}_k\right|=1\hfill \end{array}\right. $$

where *N*_*j*_ and *N*_*k*_ are the sets consisting of all neighbors of *v*_*j*_ and *v*_*k*_ at *Ti* time point in Fig. 1c, respectively.

Our method to construct DUPN is different from the work [18]. In the DUPN, we assign an uncertain value to each protein and PPI, which can distinguish the uncertain level of each protein and PPI in the dynamic PPI networks.

### Uncertain graph model

A static PPI network generally can be modeled as *G* = (*V*, *E*), where *V* = {*v*_*1*_, *v*_*2*_, …, *v*_*n*_} represents a set of proteins and *E* = {*e*_*1*_, *e*_*2*_, …, *e*_*m*_} represents a set of PPIs.

Definition 1 (Uncertain PPI network) An uncertain PPI network at *T*_*i*_ time point is defined as *UG*_*Ti*_ = (*V*_*Ti*_, *E*_*Ti*_ *,P*_*Ti*_^*V*^, *P*_*Ti*_^*E*^), where *P*_*Ti*_^*V*^: *V*_*Ti*_ → [0,1] is the function that assigns a probability of existence to each protein and *P*_*Ti*_^*E*^: *E*_*Ti*_ → [0,1] is the function that assigns a probability of existence to each PPI at *T*_*i*_ time point.

Definition 2 (Dynamic uncertain PPI network) A DUPN *DG =* {*UG*_*T1*_, *UG*_*T2*_, …, *UG*_*Tk*_}, is defined over a set of uncertain PPI networks. In Fig. 1d, the DUPN only consists three uncertain PPI networks, {*UG*_*T1*_, *UG*_*T2*_, *UG*_*T3*_}.

To deal with uncertain data, some uncertain graph models and theories [18, 23, 24] have been proposed to analyze social networks, electrical networks and biological networks and so on. In this study, we assume the probabilities of proteins and PPIs are independent. Let *G* ^*’*^_*j*_ = (*V’*_*j*_, *E’*_*j*_) denote an instantiation of an uncertain PPI network *UG*_*Ti*_ = (*V*_*Ti*_, *E*_*Ti*_ *, P*_*Ti*_^*V*^, *P*_*Ti*_^*E*^), where *V’*_*j*_ ⊆ *V*_*Ti*_ and *E’*_*j*_ ⊆ *E*_*Ti*_∩(*V’*_*j*_ × *V’*_*j*_). The instantiation is a deterministic network with an observing probability. We denote the relationship between *G* ^*’*^_*j*_ and *UG*_*Ti*_ as *UG*_*Ti*_⟹ *G* ^*’*^_*j*_. The probability of Pr(*G* ^*’*^_*j*_) is given as follows:

6 $$ {\displaystyle \begin{array}{c}\hfill \Pr \left({G}_j^{\prime}\right)=\prod \limits_{v\in {V}_j^{\prime }}{P}_{T_i}^V(v)\prod \limits_{v\in {V}_{T_i}\backslash {V}_j^{\prime }}\left(1-{P}_{T_i}^V(v)\right)\hfill \\ {}\hfill \prod \limits_{e\in {E}_j^{\prime }}{P}_{T_i}^E(e)\prod \limits_{e\in {E}_{T_i}\cap \left({V}_j^{\prime}\times {V}_j^{\prime}\right)\backslash {E}_j^{\prime }}\left(1-{P}_{T_i}^E(e)\right)\hfill \end{array}} $$

The Eq. (6) gives a probability distribution over all instantiations of the uncertain PPI network *UG*_*Ti*_ at *T*_*i*_ time point. Based on the Eq. (6), if an uncertain PPI network *UG*_*Ti*_ consists of *n* instantiations {*G* ^*’*^_*1*_, *G* ^*’*^_*2*_,…, *G* ^*’*^_*n*_}, ${\sum}_{i=1}^n\Pr \left({G}_i^{\hbox{'}}\right)=1$. In an uncertain PPI network, identifying protein complexes has to take into account all possible instantiations {*G* ^*’*^_*1*_, *G* ^*’*^_*2*_,…, *G* ^*’*^_*n*_} that are associated with the probabilities defined in Eq. (6).

Definition 3 (Expected Density) Let *UG*_*Ti*_ = (*V*_*Ti*_, *E*_*Ti*_ *, P*_*Ti*_^*V*^, *P*_*Ti*_^*E*^) denote an uncertain PPI network at *T*_*i*_ time point. *PG*_*Ti*_ = {*G* ^*’*^_*1*_, *G* ^*’*^_*2*_,…, *G* ^*’*^_*n*_} is a set of possible instantiations of *UG*_*Ti*_, where *G* ^*’*^_*j*_ = (*V’*_*Ti*_, *E’*_*Ti*_). Pr(*G* ^*’*^_*j*_) is the probability associated with instantiation *G* ^*’*^_*j*_∈*PG*_*Ti*_. Given a set of protein vertices in *UG*_*Ti*_, V_S_ ⊆ *V*_*Ti*_, the expected density of V_S_ is defined as follow:

7 $$ ED\left({V}_S,{UG}_{T_i}\right)=\frac{\sum_{j=1}^n\Pr \left({G}_j^{\prime}\right)\cdotp 2\cdotp {h}_j}{\left|{V}_S\right|\cdotp \left(\left|{V}_S\right|-1\right)} $$

where *h*_*j*_ is the number of PPIs among the proteins of V_S_ in the instantiation *G* ^*’*^_*j*_.

Definition 4 (Attached Score) Let *UG*_*Ti*_ = (*V*_*Ti*_, *E*_*Ti*_ *, P*_*Ti*_^*V*^, *P*_*Ti*_^*E*^) denote an uncertain PPI network at *T*_*i*_ time point. *PG*_*Ti*_ = {*G* ^*’*^_*1*_, *G* ^*’*^_*2*_,…, *G* ^*’*^_*n*_} is a set of possible instantiations of *UG*_*Ti*_, where *G* ^*’*^_*j*_ = (*V’*_*Ti*_, *E’*_*Ti*_). Given a set of protein vertices V_S_ ⊂ *V*_*Ti*_, a protein vertex *v*_*a*_∈*V*_*Ti*_ and *v*_*a*_∉V_S_, the attached score between *v*_*a*_ and V_S_ in the *UG*_*Ti*_, is given as follows:

8 $$ AS\left({v}_a,{V}_S\right)=\frac{\sum_{j=1}^n\Pr \left({G}_j^{\prime}\right)\cdotp {m}_j}{\left|{V}_S\right|} $$

where *m*_*j*_ is the number of PPIs between *v*_*a*_ and V_S_ in the instantiation *G* ^*’*^_*j*_.

As the uncertain graph model, an uncertain PPI network can generate a large amount of different possible instantiation. According to the Eqs. (7) and (8), the computational complexity is very high in an uncertain PPI network. Based on the studies [18, 24], the Eqs. (7) and (8) can be efficiently calculated by the Eqs. (9) and (10), respectively.

9 $$ ED\left({V}_S,{UG}_{Ti}\right)=\frac{\sum_{v_p,{v}_q\in V,{v}_p\ne {v}_q}{P}_{Ti}^E\left({v}_p,{v}_q\right)\cdotp {P}_{Ti}^V\left({v}_p\right)\cdotp {P}_{Ti}^V\left({v}_q\right)}{\left|{V}_S\right|\cdotp \left(\left|{V}_S\right|-1\right)} $$

10 $$ AS\left({v}_a,{V}_S\right)=\frac{\sum_{v_j\in {V}_S}{P}_{Ti}^E\left({v}_a,{v}_j\right)\cdotp {P}_{Ti}^V\left({v}_a\right)\cdotp {P}_{Ti}^V\left({v}_j\right)}{\left|{V}_S\right|} $$

Thus, based on the uncertain graph model, we can use the Eqs. (9) and (10) to efficiently calculate the expected density and the attached score for protein complex identification in an uncertain PPI network, respectively.

### The CDUN algorithm

Some studies has revealed the complex core-attachment organization [25]. A protein complex generally contains of a core structure and some attachment proteins. In the core structure, the proteins share high functional similarity, which are highly co-expressed [9]. The attachment proteins assist the core proteins to perform subordinate functions. Based on the core-attachment structure of protein complexes, the CDUN algorithm identifies protein complexes from all the uncertain PPI networks of a DUPN in turn. Algorithm 1 shows the pseudo-codes of the DUPN algorithm.
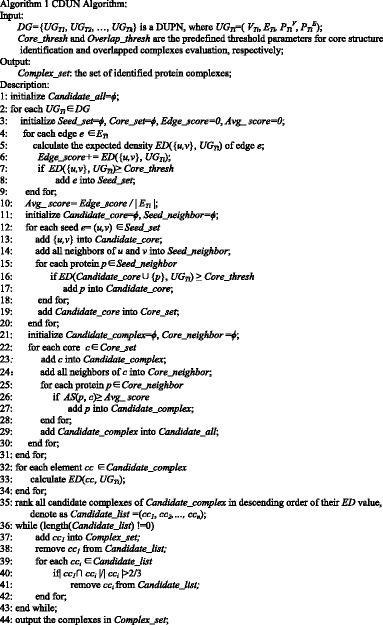


CDUN algorithm consists of two phases. CDUN firstly detects candidate protein complexes from all *UG*_*Ti*_∈*DG* in turn at line 1–31. The candidate complexes are added into *Candidate_complex* set. Then, CDUN removes the highly overlapped protein complexes from *Candidate_complex* at line 32–44, based on their *ED* value.

In the first phase, CDUN firstly calculates the expected density of all edges in *UG*_*Ti*_ based on Eq. (9) at line 4–5. *ED* ({*u,v*}, *UG*_*Ti*_) denotes the expected density of the edge between u and v. The edge will be added into *Seed_set*, if its expected density is not less than *Core_thresh* that is a predefined threshold parameter. The effect of *Core_thresh* is discussed in The effect of Core_thresh section. Average expected density of all edges is calculated at line 10. Secondly, CDUN augments each seed to generate the core structure at line 11–20. If the *ED* value of the core structure is not less than *Core_thresh*, CDUN will add the neighbor protein *p* into the core structure at line 25–28. We use the same parameter (*Core_thresh*) in lines 7 and 16 to keep the expected density of both the seeds and the core structures are not less than the *Core_thresh*. Finally, CDUN detects the attachment proteins for each core structure based on the *AS* score that is calculated by Eq. (10), and adds the attachment proteins into each core structure to form the candidate complex set *Candidate_all* at line 22–30.

The candidate protein complexes in *Candidate_all* are identified from all *UG*_*Ti*_∈*DG*, which generally overlap with each other. In the second phase, CDUN calculates the *ED* value of all candidate protein complexes in line 32–34. We rank the candidate complexes in descending order of the *ED* value (*Candidate_list =* (*cc*_*1*_, *cc*_*2*_ *,…, cc*_*n*_)) at line 35. The candidate complex with highest *ED* value in will be removed from *Candidate_list* and added into *Complex_set*. CDUN checks the overlapped degree between *cc*_*i*_ ∈*Candidate_list* and *cc*_*1*_. CDUN will removes *cc*_*i*_ from *Candidate_list* at line 39–42, if the overlapped degree is larger than the *Overlap_thresh.* In our experiments, we set the *Overlap_thresh* as 2/3. The above steps will be repeated until *Candidate_list* is empty and the final complex set *Complex_set* is generated.

## Results and discussion

### Datasets

The PPI datasets used in our experiments are the DIP [26], MIPS [27] and STRING [28] datasets, respectively. The PPI data of STRING dataset are from biomedical literature data, high-throughput data, genomic context data and co-expression data. Table 1 lists the statistics of the dataset in our experiments.

**Table 1:** The statistics of PPI datasets in experiments

High-throughput PPI data	Proteins	Interactions
DIP dataset	4928	17,491
MIPS dataset	3950	11,119
STRING dataset	5970	217,413

We download the gene expression data GSE3431 [29] from Gene Expression Omnibus, which involves 36 different time intervals. The GSE3431 consists of 3 cycles and each cycle is 12 time intervals. We calculate the average value at 12 active time points for each gene based on 3 cycles data. In our experiments, DUPN_DIP, DUPN_MIPS and DUPN_STRING are constructed based on the gene expression data GSE3431 and the PPI datasets including DIP, MIPS and STRING dataset, respectively.

To evaluate the protein complexes identified by our method, the gold standard data are CYC2008 [30]. The CYC2008 benchmark consist of 408 protein complexes, which includes some complexes of size 2. In some cases, it is hard to evaluate the performance of the methods by using the complexes of size 2. Therefore, we use 236 complexes of size more than 2 in the CYC2008 to evaluate the complexes identified in the experiments.

### Evaluation metrics

Overall, most of the complexes identification methods use two type of evaluation metrics to evaluate the performance of complexes prediction [19]. One type of evaluation metrics are precision, recall and *F*-score. The other type are sensitivity (Sn), positive predictive value (PPV) and accuracy.

Let *P(V*_*P*_ *, E*_*P*_ *)* be an identified complex and *B(V*_*B*_ *, E*_*B*_ *)* be a known complex. The neighborhood affinity score *NA(P,B)* between *P(V*_*P*_ *, E*_*P*_ *)* and *B(V*_*B*_ *, E*_*B*_ *)* is defined as follows:

11 $$ NA\left(P,B\right)=\frac{{\left|{V}_P\cap {V}_B\right|}^2}{\left|{V}_P\right|\times \left|{V}_B\right|} $$

In most studies of complex prediction, the *P(V*_*P*_ *, E*_*P*_ *)* is considered as matching the *B(V*_*B*_ *, E*_*B*_ *)* if *NA(P,B)* is larger than 0.2 [16]. In our experiments, we use the same threshold of *NA(P,B)*.

Precision, recall and *F*-score are used to evaluate of our experimental results, which are defined as follows:

12 $$ precision=\frac{N_{ci}}{Identified\_ Set} $$

13 $$ recall=\frac{N_{cb}}{\left| Benchmark\_ Set\right|} $$

14 $$ F- score=\frac{2 precision\cdot recall}{\left( precision+ recall\right)} $$

where *N*_*ci*_ and *N*_*cb*_ are the number of detected protein known complexes by our method, respectively. *Identified_Set* and *Benchmark_Set* denote the set of complexes identified by our method and gold standard dataset, respectively. In additional, we also report Sn, PPV and accuracy in our experiments. The definitions of Sn, PPV and accuracy are described in the study [16].

### The effect of *Core_thresh*

In this experiment, we evaluate the effect of the threshold parameter *Core_thresh* on the performance of CDUN. The *Core_thresh* determines not only the number of the seeds in the *Seed_set*, but also the expected density of the core structures generated from the seeds.

We use DUPN_DIP to evaluate the effect of *Core_thresh*. Table 2 shows the detailed experimental results of *Core_thresh* ranged from 0 to 1. It can be seen that when *Core_thresh* takes from 0 to 1, the number of complexes identified by our method decreases constantly. When *Core_thresh* = 0, CDUN can identify 763 protein complexes on the DUPN_DIP. It indicates that too many seeds are generated due to the value *Core_thresh* is too small. When *Core_thresh* = 1.0, CDUN cannot identify any complexes on the DUPN_DIP. It indicates that no seeds can be generated due to the value *Core_thresh* is too large. Overall, with the increase of *Core_thresh,* the precision and PPV are increased, and the recall, Sn and Accuracy are. The *F*-score of CDUN ranges from 0.246 to 0.575. When *Core_thresh* is set as 0.4, the major metrics *F*-score achieves the highest value of 0.575.

**Table 2:** The effect of *Core_thresh* on the DUPN_DIP

*Core_thresh*	#Complexes	Precision	Recall	*F*-score	Sn	PPV	Accuracy
*Core_thresh* = 0	763	0.436	0.653	0.523	**0.47**	0.648	**0.552**
*Core_thresh* = 0.1	747	0.443	**0.657**	0.53	0.461	0.649	0.547
*Core_thresh* = 0.2	651	0.493	0.619	0.549	0.443	0.667	0.544
*Core_thresh* = 0.3	551	0.55	0.589	0.569	0.433	0.66	0.535
*Core_thresh* = 0.4	433	0.6	0.551	**0.575**	0.431	0.641	0.526
*Core_thresh* = 0.5	304	0.664	0.441	0.53	0.397	0.631	0.5
*Core_thresh* = 0.6	238	0.723	0.368	0.488	0.362	0.609	0.47
*Core_thresh* = 0.7	134	0.836	0.263	0.401	0.287	0.63	0.425
*Core_thresh* = 0.8	97	**0.856**	0.199	0.323	0.201	0.689	0.372
*Core_thresh* = 0.9	56	0.839	0.144	0.246	0.128	**0.801**	0.321
*Core_thresh* = 1.0	0	–	–	–	–	–	–

The ‘#Complexes’ refers to the number of identified complexes with different *Core_thresh*. The highest value in each row is in bold

### Comparison with other methods

Then, we compare CDUN with other complex identification methods: CSO [13], Cluster ONE [12], COAN [17], CMC [8], COACH [11], HUNTER [31], MCODE [6], Transitivity Clustering method (TransClust) [32] and Spectral Clustering method (SpecClust) [33]. We test these methods on all three static PPI networks DIP, MIPS and STRING, respectively, and choose the optimal parameters. CDUN is performed on the DUPN_DIP, DUPN_MIPS and DUPN_STRING, respectively. The Table 3 lists the comparison results using CYC2008 as the benchmark.

**Table 3:** Performance comparison CDUN with other approaches using CYC2008 as benchmark

PPI Dataset	Methods	Precision	Recall	*F*-score	Sn	PPV	Accuracy
DIP	CDUN	0.6	0.551	**0.575**	0.431	0.641	0.526
	CSO	0.497	0.623	0.553	0.538	0.631	0.582
	Cluster ONE	0.337	0.441	0.382	0.378	0.696	0.513
	COAN	0.41	0.597	0.486	0.445	0.529	0.483
	COACH	0.307	0.602	0.406	0.544	0.456	0.498
	CMC	0.485	0.428	0.455	0.306	0.643	0.443
	HUNTER	**0.852**	0.119	0.208	0.164	0.644	0.325
	MCODE	0.423	0.14	0.21	0.282	0.362	0.32
	TransClust	0.13	**0.674**	0.218	**0.622**	**0.725**	**0.672**
	SpecClust	0.122	0.331	0.179	0.548	0.529	0.538
MIPS	CDUN	0.438	0.331	**0.377**	0.244	0.612	0.387
	CSO	0.391	0.344	0.365	0.283	0.641	0.426
	Cluster ONE	0.273	0.267	0.27	0.235	0.725	0.412
	COAN	0.356	0.352	0.354	0.261	0.636	0.407
	COACH	0.239	0.347	0.283	0.317	0.385	0.35
	CMC	0.335	0.322	0.328	0.361	0.468	0.411
	HUNTER	**0.538**	0.14	0.222	0.289	0.333	0.31
	MCODE	0.365	0.153	0.215	0.189	0.572	0.329
	TransClust	0.145	**0.623**	0.236	**0.544**	**0.71**	**0.621**
	SpecClust	0.095	0.182	0.125	0.41	0.37	0.389
STRING	CDUN	**0.446**	**0.674**	**0.537**	0.715	0.518	0.609
	Cluster ONE	0.13	0.343	0.188	0.671	0.494	0.569
	COACH	0.181	0.458	0.26	**0.963**	0.154	0.385
	HUNTER	0.5	0.017	0.033	0.107	0.353	0.194
	MCODE	0.079	0.131	0.099	0.681	0.257	0.418
	TransClust	0.11	0.517	0.181	0.842	**0.528**	**0.667**
	SpecClust	0.066	0.347	0.111	0.652	0.519	0.582

The highest value of each dataset is in bold. *Core_thresh* is set 0.4 for CDUN

Firstly, we use DIP dataset to compare the performance of complex detection methods. From Table 3, it can be seen that CDUN and CSO and COAN achieve the *F*-score of 0.575, 0.553 and 0.486, respectively, which significantly outperforms other methods. Both CSO and COAN exploit the GO data, which contain much valuable information related to protein complexes curated by experts. However, CDUN can achieve the highest *F*-score of 0.575 without integrating GO annotation data. HUNTER achieves the highest precision of 0.852. TransClust achieves the highest recall of 0.674, Sn of 0.622, PPV of 0.725 and accuracy of 0.672, respectively. But the precision of TransClust is only 0.13, which leads to a low *F*-score of 0.218.

Secondly, we use MIPS dataset to compare these methods. On MIPS dataset, CDUN achieves the highest *F*-score of 0.377, which are superior to other methods. HUNTER achieves the highest precision of 0.538. TransClust achieves the highest recall of 0.623, Sn of 0.544, PPV of 0.71 and accuracy of 0.621, respectively.

Thirdly, we use STRING dataset to compare these methods. STRING dataset is much larger than other two datasets. This makes more difficult for protein complex identification on STRING dataset than other two datasets. From Table 3, we can see that CDUN achieve the highest precision of 0.446, recall of 0.674 and *F*-score of 0.537, respectively. COACH achieves the highest Sn of 0.963. TransClust achieve the highest PPV of 0.528 and accuracy of 0.667, respectively. Furthermore, it can be seen that the *F*-score of other compared methods is much lower on STRING dataset than other two datasets. For instance, Cluster ONE achieves a very low *F*-score of 0.188 on STRING dataset, which is much lower than on other datasets. This is mainly because the STRING PPI network is much more complex than the PPI networks constructed by other datasets. In addition, STRING dataset integrates PPIs not only from high-throughput experiments, but also from biomedical literatures, co-expression data, genomic context data. The multiple source data generally lead to more noise data in STRING dataset. These noise data also have impact on the performance of protein complex identification methods. Compared with other methods, CDUN integrates gene expression data and STRING dataset to construct DUPN_STRING which consists of 12 uncertain PPI subnetworks, {*UG*_*T1*_, *UG*_*T2*_, *…, UG*_*T12*_. Then, CDUN identify the complexes from such uncertain PPI subnetworks. Eventually, CDUN achieve a high *F*-score of 0.537 on STRING dataset.

We also note that CDUN does not achieve high recall and accuracy in some cases. For instance, CDUN only achieve accuracy of 0.526 and 0.387 on DIP and MIPS dataset, respectively. In the future work, we will try to improve the recall and accuracy of our method further.

In additional, we compare CDUN with DCU [21] on the DIP dataset. In the study [21], the DCU method was evaluated using all the 408 complexes in the CYC2008. Therefore, we also compare CDUN with DCU using all the 408 complexes of CYC2008. The comparison results are listed in the Table 4. It can be seen that CDUN achieves higher precision and *F*-score than DCU on DIP dataset.

**Table 4:** Performance comparison CDUN with DCU on DIP Dataset

Methods	Precision	Recall	*F*-score
CDUN	0.559	0.48	0.517
DCU	0.548	0.519	0.495

### The significance of the identified complexes

In this experiment, we use GO data to evaluate biological significance of the identified complexes. The GO classifies gene product functions along biological process, molecular function and cellular component. SGD’s GO::TermFinder [34] is used to calculate the *p*-value of an identified complex with respect to GO data in our experiment. If the p-value is less than 0.01, we consider the identified complex to be statistically significant. In Table 5, We calculate the proportion of identified protein complexes with p-value less than 0.01 on the three PPI datasets.

**Table 5:** Proportion of the identified protein complexes with *p*-value less than 0.01

Datasets	Total	BP	MF	CC
DIP	433	0.947	0.801	0.904
MIPS	317	0.954	0.751	0.887
STRING	917	0.908	0.744	0.821

‘Total’ refers to the number of predicted complexes

### An study of cdc28-cyclin complexes identified by CDUN

**Fig. 2:**
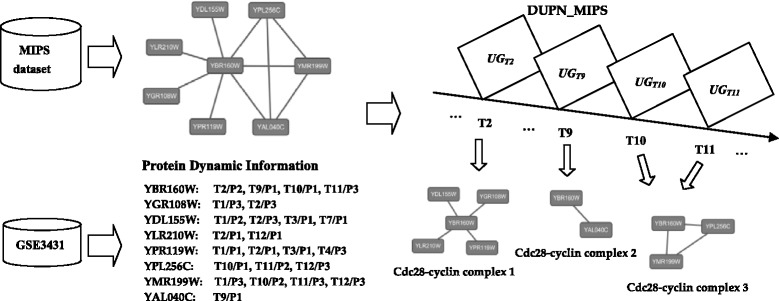
Cdc28-cyclin complexes identified by CDUN on MIPS dataset

Our method can identify many protein complexes, as well as their active time points. The cellular systems are highly dynamic and responsive to cues from the environment. These dynamic complexes results are more valuable to reveal the cellular function and organization than the static complexes results. In Fig. 2, we present an example to illustrate this.

Cdc28-cyclin complexes are a series of cyclin-dependent protein kinase holoenzyme complexes, which had been validated by [27, 35]. Cdc28-cyclin complexes consist of 10 proteins including YBR160W, YPR120C, YPL256C, YGR108W, YGR109C, YMR199W, YDL155W, YAL040C, YLR210W, YPR119W. Different Cdc28-cyclin complex contains the common kinase catalytic subunit, YBR160W, and the different regulatory cyclin partner.

The PPI networks including the 10 proteins have extracted from MIPS dataset in Fig. 2. The PPI networks don’t contain YGR109C and YPR120C, because there are no PPIs between the two proteins YGR109C and YPR120C with the other eight proteins in MIPS dataset. It is very difficult to identify the multiple Cdc28-cyclin complexes only based on the topology structure of PPI networks. Our method can use gene expression data to calculate the dynamic information of these proteins, which also have been listed in Fig. 2. From the protein dynamic information, we can see that these proteins manly are active at *T2*, *T9*, *T10* and *T11*. For instance, YBR160W, YGR108W, YDL155W, YMR199W and YLR210W are active at *T2* together. Then, our method constructs DUPN_MIPS based on PPI networks and protein dynamic information. Eventually, Cdc28-cyclin complex 1, 2 and 3 are identified from *UG*_*T2*_, *UG*_*T9*_, *UG*_*T10*_ and*UG*_*T11*_ by CDUN, which all matched in CYC2008 dataset.

From Fig. 2, we can see that the three different protein complexes are overlapped each other in the static PPI networks. Since our method constructs the DUPN, CDUN can effectively identify the three Cdc28-cyclin complexes. Furthermore, our method can identify the active time points of the three Cdc28-cyclin complexes. Cdc28-cyclin complex 1 and 2 are associated with *T2* and *T9*, respectively. Cdc28-cyclin complex 3 is associated with *T10* and *T11*. The experimental results reveal the dynamic property of Cdc28-cyclin complexes in the cellular systems. Firstly, the kinase catalytic subunit, YBR160W, associated with YGR108W, YDL155W, YMR199W and YLR210W to construct the Cdc28-cyclin complex 1 at *T2*. Then, the kinase catalytic subunit, YBR160W, associated with YAL040C to construct the Cdc28-cyclin complex 2 at *T9*. Finally, YBR160W associated with YPL256C and YMR199W to construct the Cdc28-cyclin complex 3 at *T10* and *T11*.

## Conclusions

In this paper, we firstly exploite gene expression data to calculate dynamic information of PPI networks. Then, we give a novel method to construct DUPN by integrating gene expression and PPI data based on uncertain graph theory. Next, we propose a new CDUN algorithm to detect complexes on DUPN. It is encouraging to see that our approach achieves the state-of-the-art PPI performance on different yeast PPI datasets. Furthermore, the framework of DUPN can be applied to other similar applications.

As a future study, we will collaborate with medical experts, and further analyze the dynamic property of the protein complexes identified by CDUN. We note that the recall and accuracy of our method cannot improve significantly. In the future, we will focus on this issue and try to improve the recall and accuracy of our method. In addition, we will attempt to integrate other resources, such as the TAP dataset to improve the performance of protein complex identification.

## Additional files

Additional file 1: GSE3431 gene expression data. (TXT 4397 kb)

Additional file 2: DIP PPI data. (TXT 290 kb)

Additional file 3: MIPS PPI data. (TXT 184 kb)

Additional file 4: STRING PPI data. (TXT 3624 kb)

## References

[1] Albert R, Jeong H, Barabási A-L (2000). Error and attack tolerance of complex networks. Nature.

[2] Maslov S, Sneppen K (2002). Specificity and stability in topology of protein networks. Science.

[3] Ihmels J, Friedlander G, Bergmann S, Sarig O, Ziv Y, Barkai N (2002). Revealing modular organization in the yeast transcriptional network. Nat Genet.

[4] Terentiev A, Moldogazieva N, Shaitan K (2009). Dynamic proteomics in modeling of the living cell. Protein-protein interactions. Biochem Mosc.

[5] De Las RJ, Fontanillo C (2010). Protein-protein interactions essentials: key concepts to building and analyzing interactome networks. PLoS Comput Biol.

[6] Bader GD, Hogue CW (2003). An automated method for finding molecular complexes in large protein interaction networks. BMC Bioinformatics.

[7] Srihari S, Ning K, Leong HW (2010). MCL-CAw: a refinement of MCL for detecting yeast complexes from weighted PPI networks by incorporating core-attachment structure. BMC Bioinformatics.

[8] Liu G, Wong L, Chua HN (2009). Complex discovery from weighted PPI networks. Bioinformatics.

[9] Gavin A-C, Aloy P, Grandi P, Krause R, Boesche M, Marzioch M, Rau C, Jensen LJ, Bastuck S, Dümpelfeld B (2006). Proteome survey reveals modularity of the yeast cell machinery. Nature.

[10] Leung HC, Xiang Q, Yiu S-M, Chin FY (2009). Predicting protein complexes from PPI data: a core-attachment approach. J Comput Biol.

[11] Wu M, Li X, Kwoh C-K, Ng S-K (2009). A core-attachment based method to detect protein complexes in PPI networks. BMC Bioinformatics.

[12] Nepusz T, Yu H, Paccanaro A (2012). Detecting overlapping protein complexes in protein-protein interaction networks. Nat Methods.

[13] Zhang Y, Lin H, Yang Z, Wang J, Li Y, Xu B (2013). Protein complex prediction in large ontology attributed protein-protein interaction networks. IEEE/ACM Trans Comput Biol Bioinform.

[14] Faisal FE, Milenković T (2014). Dynamic networks reveal key players in aging. Bioinformatics.

[15] Wang J, Peng X, Li M, Pan Y (2013). Construction and application of dynamic protein interaction network based on time course gene expression data. Proteomics.

[16] Li X, Wu M, Kwoh C-K, Ng S-K (2010). Computational approaches for detecting protein complexes from protein interaction networks: a survey. BMC Genomics.

[17] Zhang Y, Lin H, Yang Z, Wang J (2013). Construction of ontology augmented networks for protein complex prediction.

[18] Zhao B, Wang J, Li M, Wu F-X, Pan Y (2014). Detecting protein complexes based on uncertain graph model. IEEE/ACM Trans Comput Biol Bioinform.

[19] Przytycka TM, Singh M, Slonim DK. Toward the dynamic interactome: it's about time. Brief Bioinform. 2010;11(1):15–29. 10.1093/bib/bbp057 PMC2810115 20061351

[20] Zhang Y, Lin H, Yang Z, Wang J (2016). Construction of dynamic probabilistic protein interaction networks for protein complex identification. BMC Bioinformatics.

[21] Zhang Y, Lin H, Yang Z, Wang J. Dynamic protein complex identification in uncertain protein-protein interaction networks. In: Bioinformatics Research and Applications: 12th International Symposium. Minsk: Springer Press; 2016. p. 319.

[22] Chen B, Fan W, Liu J, Wu FX (2014). Identifying protein complexes and functional modules--from static PPI networks to dynamic PPI networks. Brief Bioinform.

[23] Yuan Y, Wang G, Chen L, Wang H (2013). Efficient Keyword Search on Uncertain Graph Data. IEEE Trans Knowl Data Eng.

[24] Zou Z, Li J, Gao H, Zhang S. Finding top-k maximal cliques in an uncertain graph. In: 2010 IEEE 26th International Conference on Data Engineering. Long Beach: IEEE press; 2010. p. 649–652.

[25] Dezső Z, Oltvai ZN, Barabási A-L (2003). Bioinformatics analysis of experimentally determined protein complexes in the yeast Saccharomyces cerevisiae. Genome Res.

[26] Xenarios I, Salwinski L, Duan XJ, Higney P, Kim S-M, Eisenberg D (2002). DIP, the Database of Interacting Proteins: a research tool for studying cellular networks of protein interactions. Nucleic Acids Res.

[27] Güldener U, Münsterkötter M, Oesterheld M, Pagel P, Ruepp A, Mewes H-W, Stümpflen V (2006). MPact: the MIPS protein interaction resource on yeast. Nucleic Acids Res.

[28] Franceschini A, Szklarczyk D, Frankild S, Kuhn M, Simonovic M, Roth A, Lin J, Minguez P, Bork P, von Mering C (2013). STRING v9. 1: protein-protein interaction networks, with increased coverage and integration. Nucleic Acids Res.

[29] Tu BP, Kudlicki A, Rowicka M, McKnight SL (2005). Logic of the yeast metabolic cycle: temporal compartmentalization of cellular processes. Science.

[30] Pu S, Wong J, Turner B, Cho E, Wodak SJ (2009). Up-to-date catalogues of yeast protein complexes. Nucleic Acids Res.

[31] Chin C-H, Chen S-H, Ho C-W, Ko M-T, Lin C-Y (2010). A hub-attachment based method to detect functional modules from confidence-scored protein interactions and expression profiles. BMC Bioinformatics.

[32] Wittkop T, Emig D, Lange S, Rahmann S, Albrecht M, Morris JH, Böcker S, Stoye J, Baumbach J (2010). Partitioning biological data with transitivity clustering. Nat Methods.

[33] Qin G, Gao L (2010). Spectral clustering for detecting protein complexes in protein–protein interaction (PPI) networks. Math Comput Model.

[34] Boyle EI, Weng S, Gollub J, Jin H, Botstein D, Cherry JM, Sherlock G (2004). GO:: TermFinder—open source software for accessing Gene Ontology information and finding significantly enriched Gene Ontology terms associated with a list of genes. Bioinformatics.

[35] Srihari S, Leong HW (2012). Temporal dynamics of protein complexes in PPI networks: a case study using yeast cell cycle dynamics. BMC Bioinformatics.

